# Chest pain in the emergency room: value of the new HEAR score identifying patients with low risk of acute coronary syndrome without troponin testing

**DOI:** 10.1016/j.clinsp.2026.101214

**Published:** 2026-10-15

**Authors:** Thibaut Pommier, Eddy Portella, Pierre Guilleminot, Adrien Goldstein, Gabriel Laurent, Charles Guenancia, Patrick Ray

**Affiliations:** a Service de Cardiologie, CHU Dijon Bourgogne, PEC2 UR 7460, Université Bourgogne Europe, Dijon, France; b Service des Urgences, CHU Dijon Bourgogne, Université Bourgogne Europe, Dijon, France

**Keywords:** Acute coronary syndrome, HEAR score, Chest pain, Emergency department

## Abstract

**Background:**

Chest pain is a frequent reason for Emergency Department (ED) visits, although only a minority of patients ultimately present an Acute Coronary Syndrome (ACS). Current diagnostic strategies rely heavily on troponin testing, but a new clinical tool requiring no blood sampling, the HEAR score, has recently been proposed. This study aimed to evaluate the performance of the HEAR score for identifying low-risk patients presenting with chest pain, using 45-day Major Adverse Cardiovascular Events (MACE) as the primary outcome.

**Methods:**

All patients presenting with chest pain to the ED of Dijon University Hospital in 2019 were retrospectively analyzed in this single-center study. Patients were stratified as “low risk” (HEAR < 2) or “high risk” (HEAR ≥ 2). Outcomes assessed at 45-days included MACE (defined as a composite of all-cause death, non-fatal myocardial infarction, and urgent coronary revascularization) and recurrence of ED consultation for chest pain.

**Results:**

Among 2177 patients presenting with chest pain, 1807 met inclusion criteria. Of these, 653 (36%) were classified as low risk (HEAR < 2). At 45-days, 22 (3%) low-risk patients reconsulted for chest pain compared with 64 (6%) in the high-risk group (p = 0.037). No MACE was identified among low-risk patients within the available follow-up data. The negative predictive value of a HEAR score < 2 was 100% (95% CI: 99.5%–100%), with an upper 95% confidence bound for the event rate of 0.46%.

**Conclusion:**

A HEAR score < 2 identifies a substantial proportion of ED chest pain patients at very low risk of short-term adverse cardiovascular events. However, given the limitations of retrospective follow-up and potential under-ascertainment of events, these findings should be considered hypothesis-generating. The HEAR score may contribute to early clinical risk stratification rather than replace troponin-based strategies.

## Highlights

• HEAR score identifies very low-risk chest pain patients.
• 36% of ED patients classified as low risk (HEAR < 2).
• No MACE observed at 45-days in low-risk group.
• Upper 95% CI for event rate: 0.46%.
• HEAR may complement troponin-based strategies.

**Clinical perspectives:**

***What is known?***

Chest pain is a leading cause of emergency department visits, although only a minority of patients are ultimately diagnosed with acute coronary syndrome. Current diagnostic strategies rely heavily on high-sensitivity troponin testing combined with clinical assessment, allowing safe rule-out of major adverse cardiovascular events with very low miss rates.

***What is new?***

In this large retrospective cohort, a HEAR score <2 identified approximately one-third of patients presenting with chest pain as being at very low risk of 45-day major adverse cardiovascular events. No events were identified in this subgroup within the available follow-up data, corresponding to a negative predictive value of 100%, although the upper bound of the 95% confidence interval suggests a non-zero residual risk.

***What are the clinical implications?***

The HEAR score may contribute to early clinical risk stratification by identifying patients with a very low likelihood of short-term adverse events. However, given the limitations of retrospective follow-up and the performance of contemporary high-sensitivity troponin-based algorithms, the HEAR score should be considered as a complementary triage tool rather than a replacement for biomarker-based strategies.

***Translational outlook:***

Prospective multicenter studies are needed to validate the safety of HEAR-based strategies in diverse clinical settings and to determine whether selected low-risk patients may be safely managed with reduced reliance on troponin testing, particularly in resource-limited environments.

## Introduction

Chest pain is one of the most common reasons for Emergency Department (ED) visits, accounting for up to 10% of consultations in France.^1^ Although only a minority of these patients ultimately present an Acute Coronary Syndrome (ACS), the need to rapidly exclude life-threatening conditions remains a major challenge for emergency physician.^2^^,^^3^ This diagnostic uncertainty contributes to high resource utilization, prolonged ED length of stay, and frequent use of complementary testing.^4^^,^^5^ Over the past decades, the evaluation of chest pain has evolved considerably, with the widespread use of cardiac biomarkers, particularly high-sensitivity troponin, and the development of clinical risk stratification tools. These strategies have significantly improved the ability to rule out ACS while reducing unnecessary hospital admissions. However, they often rely on serial blood sampling and may still contribute to ED overcrowding and increased healthcare costs.^6^ Several clinical scores have been developed to assist in the early risk stratification of patients presenting with chest pain, including the GRACE and TIMI scores.^7^^,^^8^ More recently, the HEART score has become widely adopted in emergency settings, combining clinical, electrocardiographic, and biological parameters to predict the risk of Major Adverse Cardiovascular Events (MACE).^9^, ^10^, ^11^, ^12^ Despite its strong prognostic performance, the HEART score requires troponin testing, which may delay decision-making and prolong ED stay. The HEAR score, derived from the HEART score by excluding the troponin component, has been proposed as a simplified tool to identify patients at very low risk of ACS using only clinical and electrocardiographic variables. Preliminary studies suggest that patients with a HEAR score <2 may have a very low risk of short-term adverse events, raising the possibility of a troponin-free initial triage strategy.^13^ However, data remain limited, and concerns persist regarding the safety of omitting troponin testing, particularly in routine clinical practice and outside controlled study settings. In this context, further real-world evaluations are needed. The aim of the present study was to assess the ability of a HEAR score <2 to identify patients at very low risk of 45-day major adverse cardiovascular events in a large cohort of patients presenting with chest pain to the emergency department.

## Methods

### Study design and oversight

We conducted a retrospective, single-center observational cohort study including consecutive patients presenting with chest pain to the ED of Dijon University Hospital between January 1 and December 31, 2019. The study was conducted in accordance with institutional regulations for observational research using anonymized data. Under French regulations, retrospective analyses of routine care data do not require formal ethics committee approval or a protocol number. All data were handled in compliance with the General Data Protection Regulation (GDPR). The study was conducted in accordance with the STROBE (Strengthening the Reporting of Observational Studies in Epidemiology) guidelines.

### Study population

All adult patients presenting to the ED with chest pain during the study period were screened for inclusion. Exclusion criteria were ST-segment Elevation Myocardial Infarction (STEMI) on initial Electrocardiogram (ECG), traumatic chest pain, age <18-years and patients who left the ED before medical evaluation. Clinical data, cardiovascular risk factors, past medical history, and treatments were extracted from electronic medical records (Resurgence® and DxCare® systems).

### Study objectives

The primary objective of the study was to evaluate whether a HEAR score <2 identifies patients at very low risk of short-term major adverse cardiovascular events.

### Study endpoints

The primary endpoint was the 45-day occurrence of MACE, defined as a composite of all-cause death, non-fatal myocardial infarction, and urgent coronary revascularization. Outcome ascertainment was performed through a comprehensive review of electronic medical records from the index hospital, as well as some available data from the regional emergency medical service database (SAMU), including pre-hospital care reports, discharge summaries, and inter-hospital transfers. Patients with no recorded consultation within the hospital system or no contact with the regional emergency network during the 45-day follow-up were considered as having no identified MACE within the available data. However, events occurring outside this regional network could not be captured and we did not perform active follow-up through direct patient contact, linkage with national death registries, or systematic review of records from hospitals outside the regional network.

### HEAR score assessment

The HEAR score was retrospectively calculated for each patient based on four components: history, ECG, age, and cardiovascular risk factors. All components were adjudicated using standardized definitions derived from the original HEAR score description. Investigators were blinded to outcome data at the time of score assessment. Patients were classified into two groups: low risk (HEAR score < 2) and high risk (HEAR score ≥ 2). Troponin testing was performed according to routine clinical practice and was not guided by the HEAR score (Table 1).

**Table 1:** Composition of the HEART score.

**HEART score for chest pain patients**		
**Component**	**Criteria**	**Score**
History	Highly suspicious	2
	Moderately suspicious	1
	Slightly suspicious	0
ECG	Significant ST depression	2
	Nonspecific repolarization disturbance	1
	Normal	0
Age	≥ 65-years	2
	45‒64 years	1
	< 45-years	0
Risk factors	≥ 3 risk factors or history of atherosclerotic disease	2
	1 or 2 risk factors	1
	No risk factors known	0
Troponin (Not included in the HEAR score)	> 2 × normal limit	2
	1‒2 × normal limit	1
	≤ normal limit	0
		Total

The HEAR score corresponds to the HEART score without the troponin component.

### Statistical analysis

Categorical variables are presented as counts and percentages and were compared using the Chi-Square test or Fisher’s exact test, as appropriate. Continuous variables are expressed as mean ± standard deviation or median [interquartile range], depending on distribution, and were compared using Student’s *t*-test or Mann-Whitney *U*-test.

Diagnostic performance of a HEAR score < 2 for predicting 45-day MACE was assessed by calculating the negative predictive value with 95% Confidence Intervals. Given the absence of observed events in the low-risk group, the upper bound of the 95% Confidence Interval for the event rate was estimated using the rule of three. No imputation was performed for missing data, which are reported when relevant. All tests were two-sided and a p-value < 0.05 was considered statistically significant. Analyses were performed using SPSS software (IBM SPSS Statistics, version 22).

## Results

### Study population

Between January 1 and December 31, 2019, a total of 2177 patients presented to the emergency department with chest pain. After exclusion of 370 patients (STEMI, traumatic chest pain, or absence of medical evaluation), 1807 patients were included in the analysis (Fig. 1).

**Fig. 1:**
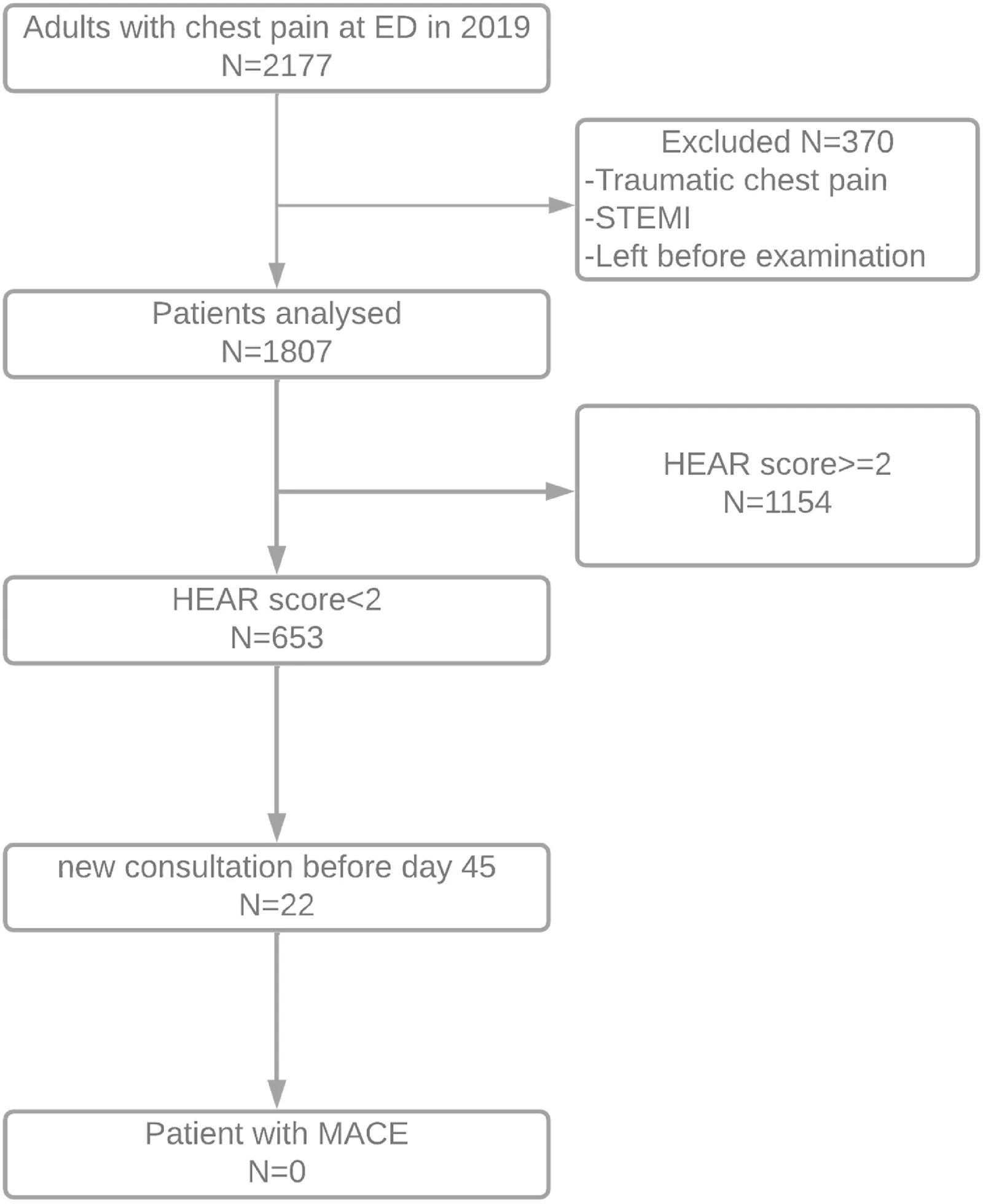
Study flowchart of patient selection.

### Baseline characteristics

The overall study population had a mean age of 51±21 years. A substantial proportion of patients presented with cardiovascular risk factors, including hypertension, smoking, and family history of coronary artery disease (Table 2). Approximately one-quarter of patients reported associated dyspnea. The mean HEART and HEAR scores were 2.7±2.1 and 2.6±1.9, respectively. Troponin levels were within the normal range in the vast majority of patients (96%), reflecting routine clinical practice in the evaluation of chest pain.

**Table 2:** Baseline characteristics of the study population.

**Characteristics**	**n = 1807**
**Risk factors**	
Age, years	51 ± 21
Obesity	121 (7%)
Hypercholesterolemia	309 (17%)
Hypertension	513 (28%)
Diabetes	189 (10%)
Smoker	408 (23%)
Family history of CAD	127 (7%)
Prior CAD	259 (14%)
**Clinical data**	
Dyspnea	434 (24%)
Palpitations	97 (5%)
Syncope	31 (2%)
Chest pain onset	
< 1 h	152 (8%)
> 1 h	876 (48%)
> 24 h	706 (39%)
Anxiety	172 (10%)
First episode	841 (47%)
**Scores**	
HEART score	2.7 ± 2.1
HEAR score	2.6 ± 1.9
HEAR Low Risk (HEAR score < 2)	653 (36%)
**History for ACS**	
Highly suspicious	443 (25%)
Moderately suspicious	455 (25%)
Slightly suspicious	909 (50%)
**ECG**	
Significant ST depression	86 (5%)
Nonspecific repolarization disturbance	211 (12%)
Normal	1213 (67%)
**Age**	
≥ 65 years	512 (28%)
45‒64 years	543 (30%)
< 45 years	752 (42%)
**Risk factors**	
≥ 3	354 (20%)
1 or 2	698 (38%)
None	755 (42%)
**Troponin**	
> 2 × normal limit	51 (3%)
1‒2 × normal limit	28 (2%)
≤ normal limit	1728 (96%)
**Emergency consultation (Day 45)**	86 (5%)

Data are presented as mean ± standard deviation or n (%). Percentages are calculated using the total study population as denominator. Missing data were not imputed and may explain discrepancies with subgroup analyses.

### HEAR score stratification

Among the 1807 patients included, 653 (36%) were classified as low risk (HEAR score < 2), while 1154 (64%) were classified as high risk (HEAR score ≥ 2) (Fig. 2). Patients in the low-risk group were significantly younger than those in the high-risk group (33±11 vs. 61±17 years, p < 0.001) and had fewer cardiovascular risk factors (Table 3). In particular, hypertension, hypercholesterolemia, diabetes, and prior coronary artery disease were markedly less frequent in low-risk patients (all p < 0.001), whereas smoking prevalence was similar between groups. Clinical presentation also differed between groups. Low-risk patients more frequently presented with a first episode of chest pain (54%vs. 42%, p < 0.001) and were more likely to have chest pain lasting longer than one day. Electrocardiograms were normal in 94% of low-risk patients compared with 73% in the high-risk group (p < 0.001). As expected, HEART and HEAR scores were significantly lower in the low-risk group (0.6 ± 0.5 vs. 3.9 ± 1.6 and 3.8 ± 1.4, respectively; p < 0.001 for both comparisons).

**Fig. 2:**
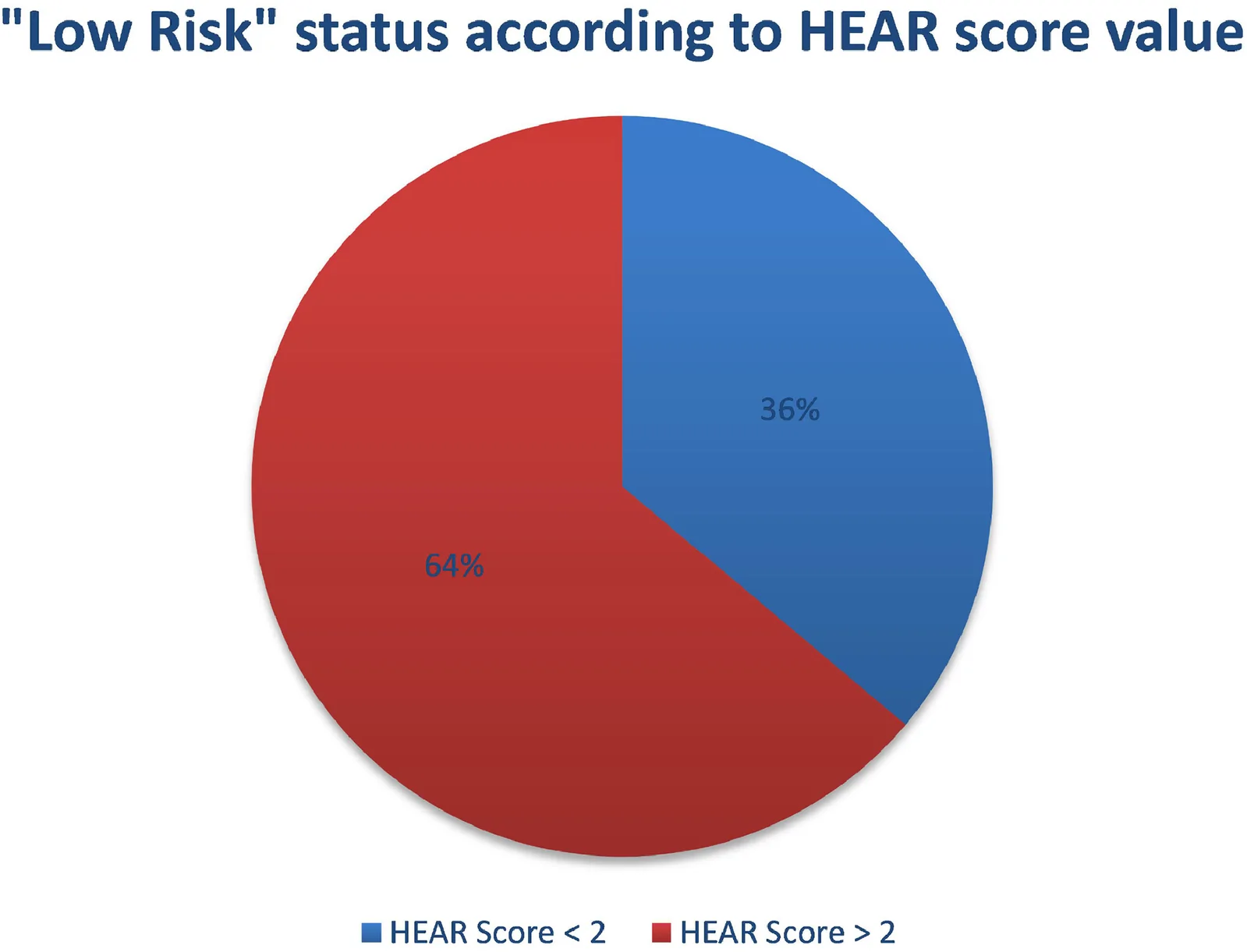
Distribution of HEAR score and classification into low- and high-risk groups.

**Table 3:** Baseline characteristics according to HEAR score categories.

	**HEAR ≧ 2**	**HEAR < 2**	**p**
	**n = 1154 (64%)**	**n = 653 (36%)**	
**Risk factors**			
Mean Age	61 ± 17	33 ± 11	<0.001
Obesity	110 (10%) [87%]	11 (2%) [94%]	<0.001
Hypercholesterolemia	303 (26%) [1%]	6 (1%) [0%]	<0.001
Hypertension	505 (44%) [1%]	8 (1%) [4%]	<0.001
Diabetes	187 (16%) [1%]	2 (0%) [4%]	<0.001
Smoker	285 (25%) [50%]	123 (19%) [61%]	0.882
Family history of CAD	107 (9%) [81%]	20 (3%) [86%]	<0.001
Prior CAD	259 (22%) [1%]	0 (0%) [5%]	<0.001
**Clinical data**			
Dyspnea	292 (25%)	142 (21%)	0.091
Palpitations	55 (5%)	42 (6%)	0.133
Syncope	24 (1%)	7 (1%)	0.112
Chest pain onset			<0.001
< 1-h	112 (10%)	40 (6%)	
> 1-h	605 (52%)	271 (41%)	
> 24-h	380 (32%)	326 (50%)	
Anxiety	93 (8%)	79 (12%)	0.005
First episode	490 (42%)	351 (54%)	<0.001
**Scores**			
HEART score	3.9 ± 1.6	0.6 ± 0.5	< 0.001
HEAR score	3.8 ± 1.4	0.6 ± 0.5	<0.001
**Clinical history suggestive of ACS**			<0.001
Highly suspicious	443 (38%)	0 (0%)	
Moderately suspicious	369 (32%)	86 (13%)	
Slightly suspicious	342 (30%)	567 (87%)	
**ECG**			<0.001
Significant ST depression	86 (7%)	0 (0%)	
Nonspecific repolarization disturbance	177 (15%)	34 (5%)	
Normal	699 (61%) [17%]	514 (79%) [16%]	
**Age**			<0.001
≥ 65-years	512 (45%)	0 (0%)	
45‒64 years	443 (38%)	100 (15%)	
< 45-years	199 (17%)	553 (85%)	
**Risk factors**			<0.001
≥ 3	354 (31%)	0 (0%)	
1 or 2	548 (47%)	150 (23%)	
None	252 (22%)	503 (77%)	
**Troponin**			<0.001
> 2 × normal limit	51 (4%)	0 (0%)	
1‒2 × normal limit	26 (2%)	2 (1%)	
≤ normal limit	1077 (94%)	651 (99%)	
**Emergency consultation (Day 45)**	64 (6%)	22 (3%)	0.037

Data are presented as mean ± standard deviation or n (%). Percentages are calculated using the total number of patients within each HEAR category. Percentages shown in brackets indicate the proportion of missing data for the corresponding variable. Comparisons were performed using Chi-Square or Mann-Whitney tests, as appropriate.

### Troponin findings

Troponin elevation was uncommon overall and occurred predominantly in the high-risk group. Elevated troponin levels (> 2 × upper limit of normal) were observed in 4% of high-risk patients compared with none in the low-risk group (p < 0.001). Only two low-risk patients had minimally elevated troponin values (1–2 × normal range).

### Clinical outcomes

At 45-days, 86 patients (5%) reconsulted the ED for chest pain, including 64 (6%) in the high-risk group and 22 (3%) in the low-risk group (p = 0.037). Among the 22 low-risk patients who reconsulted within 45-days, no MACE was identified within the available follow-up data. Reconsultations were predominantly related to non-cardiac conditions, including musculoskeletal chest pain, respiratory disorders (asthma, pneumonia, severe cough, and flu-like syndrome), or nonspecific chest pain. Electrocardiograms remained normal or unchanged in nearly all patients, and all available repeat troponin measurements were negative or within the normal range. Overall, no MACE was identified in the low-risk group (n = 653) within the available regional follow-up data (Fig. 3).

**Fig. 3:**
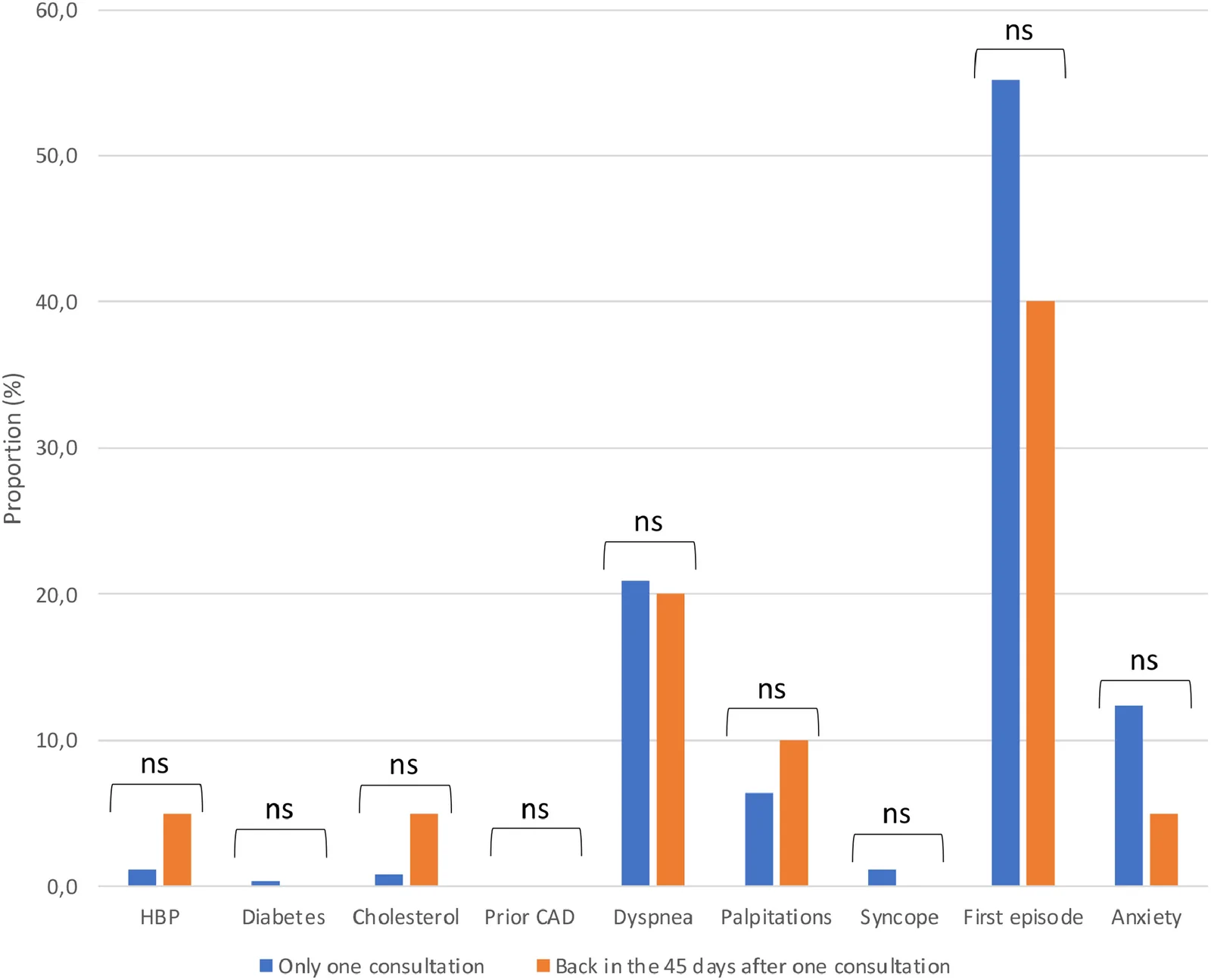
Characteristics of low-risk patients according to 45-day emergency department reconsultation.

### Diagnostic performance

The negative predictive value of a HEAR score < 2 for 45-day MACE was 100% (95% Confidence Interval [95% CI]: approximately 99.5%–100%). Given the absence of observed events in the low-risk group, the upper bound of the 95% Confidence Interval for the event rate was estimated at 0.46% using the rule of three.

## Discussion

### Main findings

In this large single-center retrospective cohort of patients presenting with chest pain to the ED, a HEAR score < 2 identified approximately one-third of patients as low risk. Within this subgroup, no MACE was identified at 45-days based on available regional follow-up data. The negative predictive value was 100%, with an upper 95% confidence bound of 0.46%, suggesting a very low but non-zero residual risk. These findings support the potential of the HEAR score as an initial risk stratification tool in patients presenting with chest pain.

### Mechanistic interpretation

The HEAR score is derived from the HEART score by removing the troponin component, thereby relying exclusively on clinical and electrocardiographic parameters. This approach assumes that a combination of non-suspicious clinical presentation, normal ECG, young age, and limited cardiovascular risk factors may be sufficient to identify patients at very low risk of ACS. In this context, our results suggest that a substantial proportion of patients presenting with chest pain could be identified early as low risk using clinical criteria alone. However, this approach does not replace biological assessment but rather reflects a potential first-step triage strategy. Importantly, the favorable prognosis observed among low-risk patients likely reflects, at least in part, their younger age and lower burden of cardiovascular risk factors, both of which are integral components of the HEAR score and contribute directly to its risk stratification performance.

### Comparison with existing literature

Our findings are consistent with previous studies evaluating the HEAR score. In a large cohort including nearly 5000 patients, Smith et al. reported a low but non-zero event rate of 30-day MACE among patients with a low HEAR score, highlighting the robustness of this threshold.^14^ This non-zero event rate, observed in larger populations, likely reflects more comprehensive event ascertainment and differences in sample size, compared with the absence of events observed in our study. Similarly, Moumneh et al. reported a low but non-zero event rate, emphasizing the importance of sample size and follow-up completeness.^15^ External validation studies have also confirmed the high sensitivity of the HEAR score for ruling out short-term MACE.^16^^,^^17^ Additional analyses suggest that most patients are not reclassified by troponin testing when HEAR is low, supporting the concept of an initial clinical triage strategy.^18^ Importantly, contemporary diagnostic strategies based on high-sensitivity troponin assays, including 0/1-h and 0/2-h rule-out algorithms, achieve miss rates below 0.2% and remain the current standard of care.^19^^,^^20^ In this context, the HEAR score should not be considered as a replacement for troponin-based strategies, but rather as a complementary tool for early clinical triage, particularly in settings where immediate troponin testing is not available. This highlights the trade-off between a purely clinical approach, which may allow faster decision-making and reduced resource utilization and healthcare costs, and biomarker-based strategies, which provide a higher level of diagnostic safety.

### Clinical implications

From a clinical perspective, the HEAR score may help identify a subset of patients with chest pain who are at very low risk of short-term adverse events. In such patients, it could potentially contribute to reducing unnecessary testing, shortening ED length of stay, and optimizing resource utilization. However, given the residual uncertainty reflected by the upper confidence interval and the limitations of retrospective event ascertainment, clinical implementation should remain cautious. In fact, this residual uncertainty should be considered in the context of contemporary high-sensitivity troponin-based rule-out strategies, which typically achieve lower miss rates. The HEAR score could be integrated into a stepwise diagnostic approach rather than used as a standalone decision-making tool. In addition, previous studies have shown a very low diagnostic yield of downstream testing, such as stress testing, in low-risk patients identified by clinical scores,^21^ supporting a possible non-invasive approach in this population. Conversely, moderate-risk patients still exhibit non-negligible event rates,^22^ highlighting the importance of avoiding overextension of low-risk strategies beyond appropriate populations.

### Study limitations

Several limitations should be acknowledged. First, the retrospective single-center design may limit the generalizability of our findings and introduces potential biases in data collection. Second, outcome ascertainment relied on hospital records and available regional emergency medical service databases. Consequently, events occurring outside this network, including out-of-hospital deaths or cardiovascular events managed in other institutions, may not have been captured, potentially resulting in under-ascertainment and underestimation of the true MACE rate. Third, the retrospective assessment of the HEAR score, particularly the “history” component, introduces subjectivity and potential inter-observer variability, and no formal assessment of inter-rater reliability was performed. Fourth, troponin testing was performed in nearly all patients as part of routine care, and therefore our study does not directly compare a HEAR-based strategy with contemporary troponin-based diagnostic algorithms. Finally, the absence of observed events in the low-risk group should be interpreted with caution. The upper bound of the 95% Confidence Interval (0.46%) suggests that the true event rate may not be negligible and may exceed the miss rates achieved by high-sensitivity troponin protocols.

## Conclusion

In conclusion, a HEAR score < 2 identifies approximately one-third of ED chest pain patients as being at very low risk of short-term MACE. While no events were identified in this subgroup within the available follow-up data, the residual uncertainty highlights the need for cautious interpretation. The HEAR score may contribute to initial clinical risk stratification, but prospective studies are required before considering its use as a standalone strategy to defer troponin testing in routine practice.

## Abbreviations

ACS, Acute Coronary Syndrome; ECG, Electrocardiogram; ED, Emergency Department; MACE, Major Adverse Cardiovascular Events; STEMI, ST-Segment Elevation Myocardial Infarction.

## Authors’ contributions

Thibaut Pommier: Conceptualization; methodology; investigation; writing-original draft; review & editing.

Eddy Portella: Data curation; investigation.

Pierre Guilleminot: Supervision; validation.

Adrien Goldstein: Supervision; validation.

Gabriel Laurent: Supervision; validation-review & editing.

Charles Guenancia: Supervision; validation-review & editing.

Patrick Ray: Conceptualization; supervision; validation-review & editing.

## Conflicts of interest

All authors declare that they have no financial relationships, personal affiliations, or other potential conflicts of interest that could have influenced the work reported in this manuscript.

## Data Availability

The datasets generated and/or analyzed during the current study are available from the corresponding author on reasonable request, in accordance with institutional and data protection regulations.
